# Choosing implementation strategies to address contextual barriers: diversity in recommendations and future directions

**DOI:** 10.1186/s13012-019-0892-4

**Published:** 2019-04-29

**Authors:** Thomas J. Waltz, Byron J. Powell, María E. Fernández, Brenton Abadie, Laura J. Damschroder

**Affiliations:** 1 Eastern Michigan University, Ypsilanti, USA; 2 Ann Arbor VA Center for Clinical Management Research, P.O. Box 130170, Ann Arbor, MI 48113-0170 USA; 3 Department of Health Policy and Management, Gillings School of Global Public Health, University of North Carolina at Chapel Hill, Chapel Hill, USA; 4 Center for Health Promotion and Prevention Research, School of Public Health, University of Texas Health Science Center at Houston, Houston, USA

**Keywords:** Consolidated Framework for Implementation Research, Expert Recommendations for Implementing Change, Implementation, Intervention mapping, Implementation strategies

## Abstract

**Background:**

A fundamental challenge of implementation is identifying contextual determinants (i.e., barriers and facilitators) and determining which implementation strategies will address them. Numerous conceptual frameworks (e.g., the Consolidated Framework for Implementation Research; CFIR) have been developed to guide the identification of contextual determinants, and compilations of implementation strategies (e.g., the Expert Recommendations for Implementing Change compilation; ERIC) have been developed which can support selection and reporting of implementation strategies. The aim of this study was to identify which ERIC implementation strategies would best address specific CFIR-based contextual barriers.

**Methods:**

Implementation researchers and practitioners were recruited to participate in an online series of tasks involving matching specific ERIC implementation strategies to specific implementation barriers. Participants were presented with brief descriptions of barriers based on CFIR construct definitions. They were asked to rank up to seven implementation strategies that would best address each barrier. Barriers were presented in a random order, and participants had the option to respond to the barrier or skip to another barrier. Participants were also asked about considerations that most influenced their choices.

**Results:**

Four hundred thirty-five invitations were emailed and 169 (39%) individuals participated. Respondents had considerable heterogeneity in opinions regarding which ERIC strategies best addressed each CFIR barrier. Across the 39 CFIR barriers, an average of 47 different ERIC strategies (SD = 4.8, range 35 to 55) was endorsed at least once for each, as being one of seven strategies that would best address the barrier. A tool was developed that allows users to specify high-priority CFIR-based barriers and receive a prioritized list of strategies based on endorsements provided by participants.

**Conclusions:**

The wide heterogeneity of endorsements obtained in this study’s task suggests that there are relatively few consistent relationships between CFIR-based barriers and ERIC implementation strategies. Despite this heterogeneity, a tool aggregating endorsements across multiple barriers can support taking a structured approach to consider a broad range of strategies given those barriers. This study’s results point to the need for a more detailed evaluation of the underlying determinants of barriers and how these determinants are addressed by strategies as part of the implementation planning process.

**Electronic supplementary material:**

The online version of this article (10.1186/s13012-019-0892-4) contains supplementary material, which is available to authorized users.

## Background

The gap between the identification of evidence-based innovations (EBIs) and their consistent and widespread use in healthcare is widely documented. Consequently, funders have prioritized implementation research dedicated to accelerating the pace of implementing EBIs in real-world healthcare settings. The challenges in implementing EBIs, or any significant organizational change, are significant and widespread. In an international survey of over 3000 executives, Meaney and Pung reported that two thirds of the respondents indicated that their companies had failed to achieve a true improvement in performance after implementing an organizational change [1]. Academic researchers can be even more critical of change efforts, noting that implementation efforts can even lead to organizational crises [2]. This is not surprising, given the lack of guidance about which implementation strategies to use for which EBIs in which contexts, and in what timeframe. Identification, development, and testing of implementation techniques and strategies, which constitute the “how to” of implementation efforts [3], are the top priorities for implementation science [4–7]. Despite the identification and categorization of a range of implementation strategies [8–10] and research assessing their effectiveness [11], there is little guidance on how to match implementation strategies with known barriers.

A foundational, though unproven, hypothesis, is that strategies must be tailored to local context [12–15]. Baker et al. [16] define tailoring as

“… strategies to improve professional practice that are planned, taking account of prospectively identified determinants of practice. Determinants of practice are factors that could influence the effectiveness of an intervention … and have been … referred to [as] barriers, obstacles, enablers, and facilitators [within the context in which the intervention occurs].”

While this overarching hypothesis seems intuitive, most studies have not tailored implementation strategies to context [17, 18]. A strategy that is successful in one context may be inert or result in failure in another. As Grol et al. state, “systematic and rigorous methods are needed to enhance the linkage between identified barriers and change strategies” [18]. Results from a systematic literature review [16] concluded that tailored implementation strategies:

“… can be effective, but the effect is variable and tends to be small to moderate. The number of studies remains small and more research is needed, including … studies to develop and investigate the components of tailoring (identification of the most important determinants, selecting interventions to address the determinants).”

Thus, tailoring strategies requires several steps: (1) assess and understand determinants within the local context, (2) identify change methods (theoretically and empirically based techniques that influence identified determinants) to address those determinants, and (3) develop or choose strategies that use those methods to address the determinants [19, 20].

Theoretical frameworks have been developed to assess potential contextual determinants, referred to as determinant frameworks [21]. The Consolidated Framework for Implementation Research (CFIR) [22] is one of the most well-operationalized [23, 24] and widely used determinant frameworks, designed specifically to systematically assess potential determinants within local settings. The CFIR includes 39 constructs (i.e., determinants), organized into five domains: Innovation Characteristics (e.g., complexity, strength of the evidence), Outer Setting (e.g., external policy and incentives), Inner Setting (e.g., organizational culture, the extent to which leaders are engaged), Characteristics of Individuals Involved (e.g., self-efficacy using the EBI in a sustainable way), and Process (e.g., planning and engaging key stakeholders). The CFIR can be used to assess context prospectively. This information can help guide decisions about the types of strategies that may be appropriate and match the needs of the local context.

Compilations of implementation strategies such as the Expert Recommendations for Implementing Change (ERIC) [10] have been developed to support systematic reporting of implementation strategies both prospectively and retrospectively. The ERIC compilation has 73 discrete implementation strategies involving one action or process. These strategies can be viewed as the building blocks of multifaceted strategies used to address the potential determinants of implementation for a specific EBI. Previous research indicates that (1) most ERIC implementation strategies are rated as high in their potential importance [25], (2) high numbers of strategies are often selected as being applicable for particular initiatives prospectively [26–28], and (3) similarly high numbers of strategies are identified in retrospective analyses [27, 29].

There is a need to identify the specific strategies stakeholders should most closely consider using when planning implementation. Which strategies to consider is likely dependent on both the specific EBI or practice innovation and the implementation context. The CFIR [22] serves as a comprehensive framework for characterizing contextual determinants of implementation while the ERIC compilation of implementation strategies serves as a comprehensive collection of discrete implementation strategies. The purpose of this study was to identify which ERIC strategies best address specific CFIR determinants, framed as barriers. The primary task of the present study involved having a wide community of implementation researchers and practitioners specify the top 7 ERIC strategies for addressing specific CFIR-based barriers. These results were then used to build a tool that may serve as an aid for considering implementation strategies based on CFIR constructs as part of a broader planning effort.

## Methods

We elicited input from experts who were part of the broad implementation science and practice communities using an online platform that instructed experts to choose which ERIC strategies would best address specific CFIR-based barriers. Experts used a ranking method because previous research found that expert stakeholders tend to rate 70% of ERIC implementation strategies generally, as “moderately important” or higher [25]. When there is a skewed distribution, in which a high number of variables are important, use of a ranking method forces respondents to compare choices relative to one another. When selecting the “top 7” strategies of a set of 73, ranking forces identification of strategies of the highest value among potential near equals.

### Recruitment

Recruitment targeted individuals familiar with CFIR, including first authors of articles citing the 2009 CFIR article, participants of a user panel for www.CFIRGuide.org (a technical assistance website), and individuals who had directly contacted the CFIR research team for assistance. Notices of the study were also distributed through the National Implementation Research Network (NIRN), the Society for Implementation Research Collaboration (SIRC), and the Implementation Networks’ message boards and mailing lists. Interested persons contacted the study team and were added to the invitation list. The research team sent reminders to potential participants weekly until at least 20 participants had provided responses to each of the 39 CFIR-based barrier statements.

### Procedure

The welcome page of the online survey provided an overview of the tasks and a link to a PDF version of the list of ERIC implementation strategies and their definitions for easy reference during the survey. The survey began with four demographic questions to capture participants’ self-reported implementation science expertise (“Implementation experts have knowledge and experience related to changing care practices, procedures, and/or systems of care. Based on the above definition, could someone accuse you of being an implementation expert?”), VA affiliation, research responsibilities, and clinical responsibilities.

**Fig. 1:**
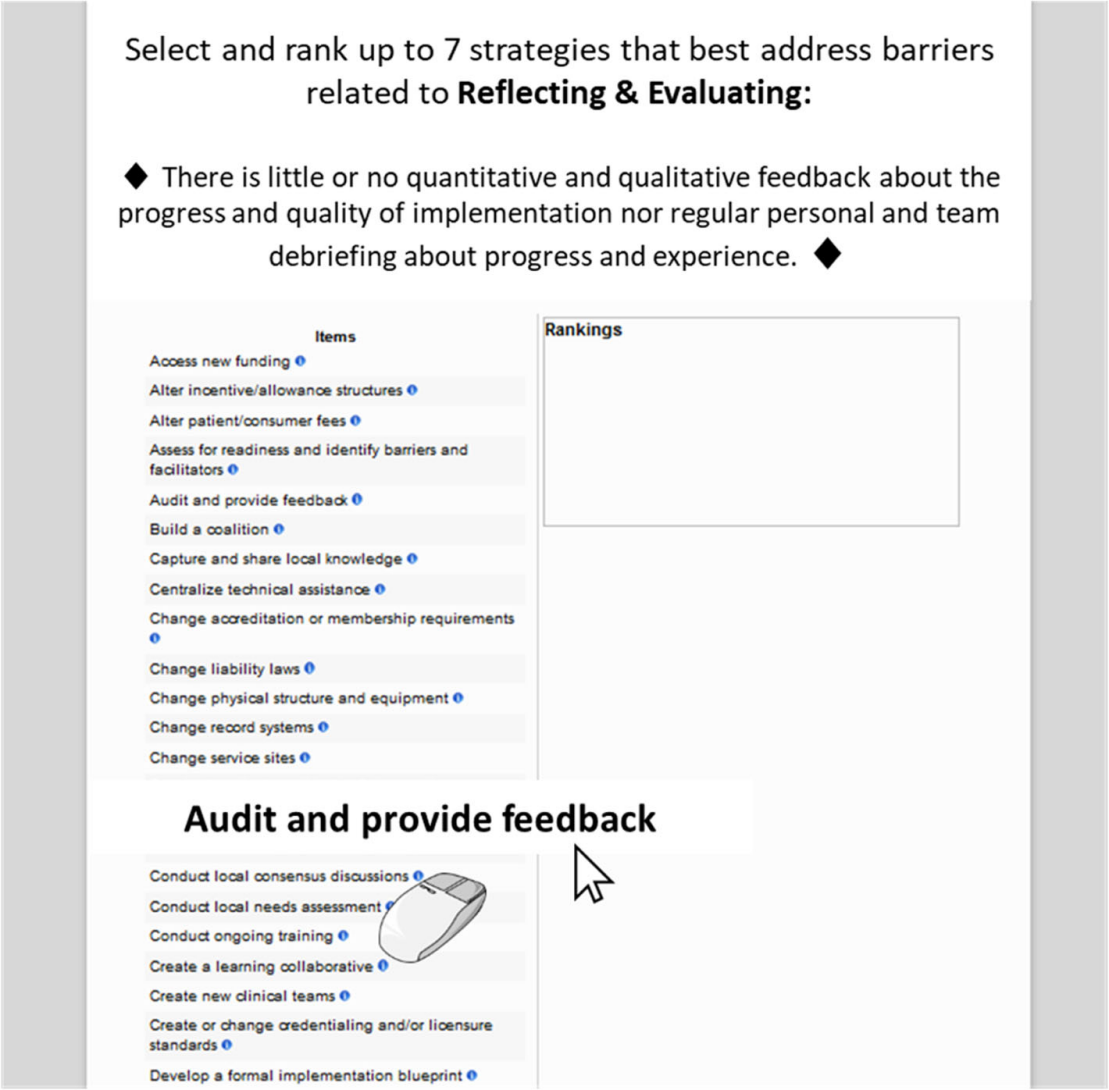
Screenshot of the ranking task. Participants were instructed to drag and drop strategies from the left-hand column to the ranking box on the right. Once placed in the ranking box, the relative position of the strategies could be manipulated

The second section of the survey presented participants with a randomly selected CFIR barrier (the 39 CFIR constructs were each used to describe a barrier) and a brief barrier narrative (see Table 2). Participants could choose to skip or accept the barrier presented. If the participant selected “skip,” they could choose to (a) receive another randomly selected CFIR barrier (selection without replacement), (b) go to the final five questions of the survey, or (c) exit with the option to return to the survey at a more convenient time. If the barrier was accepted, the participant was taken to a new page where they were instructed to “Select and rank up to 7 strategies that would best address the following barrier.” The CFIR barrier and its accompanying description were displayed, followed by further instructions to “Drag and drop ERIC strategies from the left column to the Ranking box and order them so that #1 is the top strategy” (see Fig. 1). The left column listed all 73 ERIC discrete implementation strategies. Next to each strategy was an information icon that users could click to see a pop-up definition of the strategy. These definitions were identical to those published [10] and provided in the PDF at the beginning of the survey. Participants had unlimited time to select their strategies and place them in rank order. The bottom of the page provided an optional open comment section to add an explanation, rationale, or information about their confidence in their ranking. Responses were saved when the participants clicked on the “Next” button, after which another randomly selected CFIR-based barrier was presented that the participant could either skip or accept. The option to skip or accept CFIR categories was given to ensure respondents could opt out of providing responses if they were unfamiliar with a particular barrier. Participants were encouraged to provide responses for at least eight CFIR barriers.

The third and final section of the survey asked participants to report to what extent feasibility, improvement opportunity, validity, difficulty, and relevance of each ERIC strategy influenced their ranked choices, on a three-point scale from “not influential” to “extremely influential.”

## Results

### Participants

A total of 435 invitations were emailed, and 169 individuals provided responses (39% participation rate) including demographics. Most participants self-identified as an implementation expert (85.2%). A similar percentage (81.7%) indicated that more than 50% of their employment was dedicated to research. Only 3% indicated that none of their employment was dedicated to research and the remainder (15.4%) indicated less than 50% of their employment was dedicated to research. About one fourth of the participants had clinical responsibilities (26.6%) with 7.1% providing direct patient care, 12.4% serving a management or oversight role for direct care, and 7.1% indicating having both direct care and management responsibilities. About a third (34.3%) of the participants were either employed or affiliated with the US Veterans Health Administration (VHA). Most respondents were from the USA (83%), Canada (7%), and Australia (4%), with the remainder from British, European, and African countries.

Each participant provided strategy rankings for an average of 6.1 CFIR barriers (range 1–36). The number of respondents for each CFIR barrier varied; across the barriers, an average of 26 respondents (range 21 to 33) selected up to seven strategies they felt would best address that barrier.

### Endorsement of strategies

Participants identified an average of six strategies (SD = 4.75, range = 1 to 7, mode = 7) per barrier. There was considerable heterogeneity of opinion regarding which ERIC strategies best addressed each CFIR barrier. Across all 39 CFIR barriers, an average of 47 different ERIC strategies (SD = 4.8; range 35 to 55) was endorsed at least once, as being one of the top 7 strategies that would best address each barrier. Considering the 39 CFIR barriers and 73 ERIC strategies, there were 2847 possible barrier-strategy combinations (i.e., 39 CFIR barriers X 73 ERIC strategies). Altogether, at least one participant endorsed 1832 (64%) of the 2847 possible individual barrier-strategy combinations.

Table 1 shows the distribution of endorsements for a single example CFIR barrier, Reflecting and Evaluating (within the Process domain), which was described as, “There is little or no quantitative and qualitative feedback about the progress and quality of implementation nor regular personal and team debriefing about progress and experience.” Twenty-five participants identified ERIC strategies to address this barrier; 43 ERIC strategies were endorsed by at least one of the 25 respondents.

**Table 1:** ERIC strategies endorsed to address CFIR barrier: Reflecting and Evaluating

Level*	ERIC strategy	Endorsements	
		*n* = 25 respondents	
		Count	Percentage (%)
1	Develop and implement tools for quality monitoring	15	60
1	Audit and provide feedback	14	56
2	Develop and organize quality monitoring systems	10	40
2	Facilitate relay of clinical data to providers	9	36
2	Obtain and use patients/consumers and family feedback	7	28
2	Organize clinician implementation team meetings	7	28
2	Purposely reexamine the implementation	7	28
2	Use data experts	7	28
2	Capture and share local knowledge	6	24
2	Facilitation	5	20
	Change record system	4	16
	Conduct ongoing training	4	16
	Develop a formal implementation blueprint	4	16
	Provide local technical assistance	4	16
	Assess for readiness and identify barriers and facilitators	3	12
	Conduct cyclical small tests of change	3	12
	Tailor strategies	3	12
	Use an implementation adviser	3	12
	Use data warehousing techniques	3	12
	Use train the trainer strategies	3	12
	Build a coalition	2	8
	Conduct local consensus discussions	2	8
	Create a learning collaborative	2	8
	Identify and prepare champions	2	8
	Inform local opinion leaders	2	8
	Involve executive boards	2	8
	Prepare patients/consumers to be active participants	2	8
	Provide ongoing consultation	2	8
	Recruit, designate, and train for leadership	2	8
	Conduct educational meetings	1	4
	Conduct educational outreach visits	1	4
	Conduct a local needs assessment	1	4
	Develop academic partnerships	1	4
	Develop disincentives	1	4
	Intervene with patients/consumers to enhance uptake and adherence	1	4
	Involve patients/consumers and family members	1	4
	Place innovation on a fee for service lists/formularies	1	4
	Provide clinical supervision	1	4
	Revise professional roles	1	4
	Stage implementation scale up	1	4
	Start a dissemination organization	1	4
	Use capitated payments	1	4
	Work with educational institutions	1	4

*Note:* ERIC strategies selected when respondents were asked to “… select … top 7 …” to address the following barrier: “There is little or no quantitative and qualitative feedback about the progress and quality of implementation nor regular personal and team debriefing about progress and experience”

*Level of endorsement: Level 1 includes strategies endorsed by at least 50% of respondents. Level 2 includes strategies endorsed by 20–49.9% of respondents

Over half of the 25 respondents endorsed two strategies (“Develop and implement tools for quality monitoring” and “Audit and Provide Feedback”). We characterize these strategies as level 1 endorsements because the majority of respondents endorsed those strategies. The top quartile of endorsement levels across all individual barrier-strategy combinations included combinations with at least 20% of respondents endorsing that combination. We refer to strategies with 20–49.9% endorsement as “level 2” endorsements. Table 1 lists eight level 2 strategies chosen by the participants that would best address the barrier related to lack of Reflecting and Evaluating.

Overall, level 2 strategies comprised 332 (18.1%) of the 1832 endorsed barrier-strategy combinations while level 1 comprised 39 (2.1%). Twenty-one of the 39 CFIR barriers did not have a level 1 recommended strategy; Level 1 endorsements ranged from 0 to 3. CFIR barriers had a range of 5 to 15 level 2 strategies endorsed by respondents. Table 2 lists the number of level 1 and level 2 endorsements for each CFIR barrier. The table includes the barrier description used for each CFIR construct. Additional file 1 lists the level of endorsement for each of the 2847 possible individual barrier-strategy combinations.

**Table 2:** Number of level 1 and level 2 strategies by CFIR barrier

CFIR construct	Barrier description	No. ERIC strategies	
		Level 1	Level 2
Intervention source	Stakeholders have a negative perception of the innovation because of the entity that developed it and/or where it was developed.	0	9
Evidence strength and quality	Stakeholders have a negative perception of the quality and validity of evidence supporting the intervention.	0	10
Relative advantage	Stakeholders do not see the advantage of implementing the innovation compared to an alternative solution or keeping things the same.	0	11
Adaptability	Stakeholders do not believe that the innovation can be sufficiently adapted, tailored, or re-invented to meet local needs.	1	10
Trialability	Stakeholders believe they cannot test the innovation on a smaller scale within the organization or undo implementation if needed.	0	10
Complexity	Stakeholders believe that the innovation is complex based on their perception of duration, scope, radicalness, disruptiveness, centrality, and/or intricacy and number of steps needed to implement.	0	15
Design quality and packaging	Stakeholders believe the innovation is poor quality based on the way it is bundled, presented, and/or assembled.	0	7
Cost	Stakeholders believe the innovation costs and/or the costs to implement (including investment, supply, and opportunity costs) are too high.	1	9
Patient needs and resources	Patient needs, including barriers and facilitators to meet those needs, are not accurately known and/or this information is not a high priority for the organization.	3	5
Cosmopolitanism	The organization is not well networked with external organizations.	3	7
Peer pressure	There is little pressure to implement the innovation because other key peers or competing organizations have not already implemented the innovation nor is the organization doing this in a bid for a competitive edge.	0	8
External policy and incentives	External policies, regulations (governmental or other central entity), mandates, recommendations or guidelines, pay-for-performance, collaborative, or public or benchmark reporting do not exist or they undermine efforts to implement the innovation.	0	7
Structural characteristics	The social architecture, age, maturity, and size of an organization hinder implementation.	0	9
Networks and communications	The organization has poor quality or non-productive social networks and/or ineffective formal and informal communications.	2	7
Culture	Cultural norms, values, and basic assumptions of the organization hinder implementation.	1	12
Implementation climate	There is little capacity for change, low receptivity, and no expectation that the use of the innovation will be rewarded, supported, or expected.	1	6
Tension for change	Stakeholders do not see the current situation as intolerable nor do not believe they need to implement the innovation.	0	8
Compatibility	The innovation does not fit well with existing workflows nor with the meaning and values attached to the innovation, nor does it align well with stakeholders’ own needs and/or it heightens the risk for stakeholders.	0	10
Relative priority	Stakeholders perceive that the implementation of the innovation takes a backseat to other initiatives or activities.	0	6
Organizational incentives and rewards	There are no tangible (e.g., goal-sharing awards, performance reviews, promotions, salary raises) or less tangible (e.g., increased stature or respect) incentives in place for implementing the innovation.	1	7
Goals and feedback	Goals are not clearly communicated or acted upon, nor do stakeholders receive feedback that is aligned with goals.	1	6
Learning climate	The organization has a climate where (a) leaders do not express their own fallibility or need for stakeholders’ assistance or input; (b) stakeholders do not feel that they are essential, valued, and knowledgeable partners in the implementation process; (c) stakeholders do not feel psychologically safe to try new methods; and (d) there is not sufficient time and space for reflective thinking or evaluation.	1	6
Readiness for implementation	There are few tangible and immediate indicators of organizational readiness and commitment to implement the innovation.	1	6
Leadership engagement	Key organizational leaders or managers do not exhibit commitment and are not involved, nor are they held accountable for the implementation of the innovation.	0	9
Available resources	Resources (e.g., money, physical space, dedicated time) are insufficient to support the implementation of the innovation.	1	7
Access to knowledge and information	Stakeholders do not have adequate access to digestible information and knowledge about the innovation nor how to incorporate it into work tasks.	3	7
Knowledge and beliefs about the intervention	Stakeholders have negative attitudes toward the innovation, they place low value on implementing the innovation, and/or they are not familiar with facts, truths, and principles about the innovation.	1	11
Self-efficacy	Stakeholders do not have confidence in their capabilities to execute courses of action to achieve implementation goals.	0	12
Individual stage of change	Stakeholders are not skilled or enthusiastic about using the innovation in a sustained way.	0	12
Individual identification with organization	Stakeholders are not satisfied with and have a low level of commitment to their organization.	0	9
Planning	A scheme or sequence of tasks necessary to implement the intervention has not been developed or the quality is poor.	2	6
Opinion leaders	Opinion leaders (individuals who have a formal or informal influence on the attitudes and beliefs of their colleagues with respect to implementing the intervention) are not involved or supportive.	2	6
Formally appointed internal implementation leaders	A skilled implementation leader (coordinator, project manager, or team leader), with the responsibility to lead the implementation of the innovation, has not been formally appointed or recognized within the organization.	1	11
Champions	Individuals acting as champions who support, market, or “drive-through” implementation in a way that helps to overcome indifference or resistance by key stakeholders are not involved or supportive.	1	6
External change agents	Individuals from an outside entity formally facilitating decisions to help move implementation forward are not involved or supportive.	0	10
Key stakeholders	Multifaceted strategies to attract and involve key stakeholders in implementing or using the innovation (e.g., through social marketing, education, role modeling, training) are ineffective or non-existent.	1	9
Patients/customers	Multifaceted strategies to attract and involve patients/customers in implementing or using the innovation (e.g., through social marketing, education, role modeling, training) are ineffective or non-existent.	3	6
Executing	Implementation activities are not being done according to plan.	0	12
Reflecting and evaluating	There is little or no quantitative and qualitative feedback about the progress and quality of implementation nor regular personal and team debriefing about progress and experience.	2	8

*Note*: Level 1 includes strategies endorsed by at least 50% of respondents. Level 2 includes strategies endorsed by 20–49.9% of respondents (top quartile of endorsements)

Table 3 summarizes considerations that influenced participants’ selections of strategies. The relevance of the strategy to the CFIR barrier was the single most influential consideration followed by the perceived improvement opportunity and feasibility of the chosen strategies. Level of difficulty, while endorsed by the majority of respondents as extremely or somewhat influential, nonetheless, had the lowest level of endorsement.

**Table 3:** Variables influencing ranking

	Not influential (%)	Somewhat influential (%)	Extremely influential (%)
Relevance (Does the strategy have direct relevance to the barrier?)	0.0	14.8	85.2
Improvement opportunity (Will this strategy make a big impact?)	1.6	34.4	63.9
Feasibility (Can the strategy realistically be applied to the barrier?)	8.2	38.5	53.3
Validity (Is the evidence base for the strategy compelling?)	13.1	62.3	24.6
Level of difficulty (What are the work and resource requirements for the strategy?	27.9	51.6	20.5

*Note*: *n* = 122 (72.2% response rate)

Of the 169 participants, 73 (43%) provided comments in the optional comment boxes at the bottom of each ranking page; a total of 187 comments were provided. Comments were coded by themes that were inductively derived based on descriptive coding of content [30]. Coding was completed by the primary coder (BA) and then reviewed by the research team. These themes included confidence in choice, elaboration on choice, issues related to CFIR barrier, issues related to context, issues related to implementation strategies, technical issues with the survey platform, and nonspecific comments. Twenty-three of the comments were coded as reflecting multiple themes. Most comments involved participants elaborating on their choices. Table 4 provides a summary of the qualitative content.

**Table 4:** Themes derived from qualitative responses to open-ended comment boxes

Theme	Example quotes	%
Confidence in choices	I again have low confidence in my selections as a functional analysis of the low opinion [Evidence Strength & Quality] would be required to identify strategies to address. Difficult to only choose 7. I have moderate confidence in my ranking.	12
Elaborating on choices	I think the items I chose are fairly strong supporters of adaptability. I think adaptability is dependent upon facilitation of inventiveness/adaptability by someone who knows both the setting and the intervention. Revising professionals’ roles supports adaptability, however, there is often such a struggle with understanding team roles, even without revision, this needs constant vigilance. This order of steps presumes that there is already someone who is familiar with the organization and intervention who could be trained for leadership within a relatively short time. Items 1 and 2 would be reversed if there are no clear candidates who could be recruited and trained to be an implementation leader, and this process would be facilitated by having an implementation blueprint in place.	62
Issues related to CFIR barrier	This barrier is hard to envision, too. What exactly is the barrier? Is there an identified “champion” who is not supportive, creating passive resistance, or perhaps thinks he is helping but is actually hampering progress? Or is there just no champion. Again, involves very different processes. It’s not entirely clear to me what this barrier description means. I can read it several ways. Is there a practice, owned by a hospital, that is trying to implement a change but the hospital’s staff is not helping, or is actively opposed? Or is this an organization that should be getting change management help from, for example, a regional extension center but the REC is just ignoring them? / / Lacking more detail, I approached this from the perspective that the outside entity is unhelpful so the organization has to compensate internally.	5
Issues related to context	The approach for these detail-less exercises would be easier to develop with more context or specific situation. … it seems to me that unless one understands the factors that contribute to the lack of incentives, it is not possible to recommend with any confidence the utility of different strategies to address the issue. Similarly, the phase of implementation at which this problem is observed would have implications for the strategies likely to be effective. For example, at the early exploration phase, the extent to which such incentives, concrete or symbolic, could be cultivated in the organization and service system would be a point of discussion. Alternatively, if an innovative practice is implemented but does not reach sustainment, and the lack of incentives is identified as a factor contributing to the lack of sustainment, then the strategies to address the problem would likely differ.	7
Issues related to implementation strategies	Behavioral change strategies targeting motivation are lacking in the ERIC list. Not sure any of these strategies would rectify poor communication and networks.	10
Technical issues	Website would not let me reorder this for some reason. Just an fyi. The order indicated here is close enough. The drag and drop functionality is super hard to work with in this survey. I have done my best on rank ordering but OY! What a pain!	3
Nonspecific comments	OK Proverbs 29:18. When there is no vision, the people perish. [peer pressure]	1

*Note:* Percentages reflect the percentage of comments received. Comments were received for 18.2% of responses (of 1030 opportunities). Comments were provided by 73 of the 169 participants (43.2%) for one or more barriers

## Discussion

This is the first study to engage expert implementation stakeholders in identifying discrete ERIC implementation strategies that would best address specific barriers based on the 39 CFIR contextual determinants of implementation. Of the 39 CFIR barrier scenarios, 21 barriers had at least one and up to three ERIC strategies chosen as “in the top seven” by most participants (i.e., level 1 endorsements). Otherwise, there was substantial heterogeneity in strategies chosen to address barriers. A range of 35 to 55 strategies was endorsed by one or more respondents across the range of barriers. These results illustrate that with the exception of a few strategies, there is little consensus regarding which strategies are most appropriate to address CFIR barriers when experts used a ranking approach.

Previous research reported that most ERIC strategies (i.e., 54 of 73 or 74.0%) were rated as moderately to extremely important by participating experts [25]. In the current study, of the 22 ERIC strategies receiving level 1 endorsement, 16 (72.7%) had been rated in the top quadrant of importance and feasibility out of the list of 73 strategies [25]. In contrast, only two of the 22 strategies (9.1%) were rated in the lowest quadrant of both importance and feasibility. Thus, the present findings indicate that importance and feasibility may vary based on the presence of specific CFIR barriers.

The present results can be juxtaposed with those of Rogal and colleagues [29] where a retrospective analysis of ERIC strategies used to implement a new generation EBI for hepatitis C virus (HCV) across 80 VHA health stations was conducted. Respondents (stakeholders actively involved in the HCV EBI’s implementation) were presented with the list of ERIC strategies and asked, “Did you use X strategy to promote HCV care in your center?” Respondents indicated yes for an average of 25 strategies (SD = 14) with higher numbers of strategies being associated with higher numbers of new EBI treatment starts. Thus, the heterogeneity of ERIC strategies endorsed in the present study based on brief descriptions of barriers, independent of a specific EBI implementation, is consistent with the heterogeneity observed in Rogal et al.’s retrospective assessment of HCV EBI implementation. Similar results have been found in other healthcare sectors as well [27, 28].

Based on results from the current study, we developed the CFIR-ERIC Implementation Strategy Matching Tool, which is publicly available on www.cfirguide.org. Because of the wide diversity of responses by our expert respondents and the lack of consensus this represents for the majority of endorsements, this tool must be used with caution.

It is likely that the diversity of endorsements of specific implementation strategies to address certain barriers also reflects the diversity of assumptions about how barriers interact with the program-specific needs experienced by the respondents. Another source of diversity for the endorsements is likely due to differences in interpretation of both the barrier described and the strategy. Nevertheless, these nascent results provide a productive starting point for structuring planning for tailoring an implementation effort to known contextual barriers. As more implementation researchers document barriers and strategies using these frameworks, and as processes for the development and selection of implementation strategies become more widespread [19, 20], the knowledge base for a barrier-strategy mapping scheme will strengthen.

### Example application of the CFIR-ERIC tool

To illustrate the potential value of these findings, we provide a case example based on a published implementation evaluation of a telephone lifestyle coaching program (TLC) within VHA [31]. This study evaluated CFIR constructs across 11 medical centers that had varying success in implementing the program. Implementation effectiveness was assessed by measuring penetration, defined as “integration of a practice within a service setting” [32], and was operationalized as the rate of Veteran patients who were referred to TLC, divided by the number of Veterans who were candidate beneficiaries of the program. The site with the highest penetration had a rate of referrals that was more than seven times higher than the lowest penetration site. Seven CFIR constructs were identified as being associated with outcomes—in nearly all cases, these were rated as barriers at sites with lower penetration. One core theory within implementation science is that implementation will have the highest likelihood for success if implementation strategies are selected based on an assessment of context, all else being equal. In the case of TLC implementation, implementation strategies could be selected to address the specific barriers identified and packaged as a multi-level, multifaceted set of supports to implement TLC in medical centers.

The CFIR-ERIC Mapping Tool could be used to generate a list of ERIC strategies to consider for addressing each CFIR barrier. In this example, all 73 ERIC strategies received at least one endorsement across the seven CFIR barriers associated with TLC outcomes. The top 37 strategies are presented in Table 5, listing only strategies with a level 1 or 2 recommendation for at least one of the seven CFIR barriers. This list highlights the heterogeneity and thus nascent nature of the tool. However, it provides a useful starting point. Strategies are ordered by “cumulative percent”; the top listed strategies are ones that have the highest cumulative level of endorsement across all seven CFIR barriers. For example, Identify and Prepare Champions, is the first listed strategy. This means that using this strategy has the highest likelihood of addressing facets of one or more barriers; in fact, it was a level 1 or 2 endorsement for six of the seven barriers. Thus, a single strategy may simultaneously address multiple barriers, depending on how it is operationalized. Six of the strategies listed in Table 5 have level 1 endorsements (indicated by bolding) but are not all positioned at the top of the list. Level 1 strategies are highly specific to individual CFIR barriers and merit close reflection, independent of the cumulative percentages. This example illustrates how the CFIR-ERIC Mapping Tool can be used to support broad consideration of implementation strategies based on the assessment of contextual barriers.

**Table 5:** Telephone lifestyle coaching case example

ERIC strategies	Cumulative percent (%)	Structural characteristics (%)	Networks and communications (%)	Compatibility	Organizational incentives and rewards (%)	Planning	Formally appointed internal implementation leaders (%)	Key stakeholders (%)
Identify and prepare champions	**248**	***27***	17	***21***	***25***	***31***	*64*	*63*
Assess for readiness and identify barriers and facilitators	**205**	**36**	13	***34***	13	***42***	***29***	***38***
Develop a formal implementation blueprint	**171**	18	13	3	8	*73*	***46***	8
Conduct local consensus discussions	**164**	14	***22***	***41***	8	***23***	14	***42***
Build a coalition	**143**	***27***	***39***	***21***	17	4	11	***25***
Conduct local needs assessment	**137**	18	9	***21***	8	*50*	11	***21***
Alter incentive/allowance structures	**135**	18	0	10	*71*	12	7	17
Create a learning collaborative	**135**	18	***35***	14	13	8	14	***33***
Organize clinician implementation team meetings	**129**	14	*52*	14	8	15	***21***	4
Facilitation	**125**	9	***26***	***24***	4	***23***	***21***	17
Recruit, designate, and train for leadership	**120**	18	17	0	***21***	12	***39***	13
Inform local opinion leaders	**113**	14	***22***	3	17	0	***29***	*29*
Identify early adopters	**112**	***23***	17	10	13	12	***25***	13
Promote network weaving	**111**	***23***	*57*	0	8	4	7	13
Tailor strategies	**105**	18	4	***38***	17	12	0	17
Capture and share local knowledge	**102**	***23***	***26***	14	8	15	4	13
Conduct cyclical small tests of change	**102**	*2* ***3***	9	***38***	13	12	0	8
Promote adaptability	**88**	***23***	0	***45***	4	0	0	17
Use advisory boards and workgroups	**87**	5	13	3	4	15	***21***	*2* ***5***
Involve executive boards	**81**	14	9	3	13	0	18	***25***
Use an implementation adviser	**72**	5	9	10	0	15	***29***	4
Develop and implement tools for quality monitoring	**71**	5	0	3	***21***	***31***	7	4
Access new funding	**69**	5	4	3	***38***	8	7	4
Centralize technical assistance	**67**	5	***26***	10	0	12	11	4
Obtain formal commitments	**67**	9	9	0	13	4	***29***	4
Provide local technical assistance	**64**	18	9	14	0	15	4	4
Purposely reexamine the implementation	**63**	0	4	***28***	4	15	4	8
Revise professional roles	**59**	18	0	10	13	0	18	0
Fund and contract for clinical innovation	**57**	14	0	10	***21***	0	4	8
Conduct educational meetings	**56**	5	13	10	0	8	0	***21***
Audit and provide feedback	**54**	5	17	7	***21***	0	0	4
Involve patients/consumers and family members	**52**	9	9	10	4	4	4	13
Stage implementation scale up	**51**	14	0	10	4	15	7	0
Provide ongoing consultation	**50**	9	0	3	4	4	***21***	8
Change physical structure and equipment	43	**32**	0	7	4	0	0	0
Conduct ongoing training	32	0	4	0	0	***23***	0	4
Use other payment schemes	25	0	0	0	***25***	0	0	0

*Note:* Level 1 endorsements are in bold. Percentages for level 1 endorsements for each CFIR barrier are in italics. Level 2 endorsements are in bold italics

There are several possible explanations for the reason respondents lacked consensus on their endorsed strategies, even when presented with seemingly specific contextual barriers. First, respondents had experience in implementation (practice or evaluation) in diverse settings around the world. Though CFIR constructs have been rated as highly operationalized [23], the definitions are nonetheless, designed to be relatively abstract so that they can be applied across a range of settings and to align with higher level theories that underlie each construct. Additionally, while the barriers described a specific challenge, they did not elucidate why the challenge was occurring which can be the most important information needed to inform strategy selection. Therefore, the respondent had to make assumptions about how exactly a particular “barrier” arose based on a specific determinant or combination of determinants (low knowledge, skills, capacity, negative attitudes, etc.). These interpretations of cause likely influenced opinions about which strategies would best address each barrier. Second, ERIC strategies have a range of specificity. For example, a strategy of identifying and preparing a champion is relatively discrete and clear but facilitation is a process that draws on a broad range of additional strategies. Additionally, strategies likely comprise either single or multiple mechanisms of change [33]. The ERIC strategies do not include descriptions of the mechanism of change (i.e., why it works), though work to link these strategies to potential mechanisms is currently underway [34, 35]. Thus, respondents may draw on a wide range of assumptions about how each strategy’s mechanisms may address lower-level determinants associated with each CFIR barrier. Taking all these considerations into account leads to the highly likely proposition that respondents each chose strategies based on an ideographic array of underlying assumptions. It is also plausible that multiple strategies and strategy combinations can, in fact, be used to successfully address determinants, indicating equifinality (i.e., multiple pathways) in producing positive implementation outcomes [36]. Thus, future research needs to elicit greater detail from respondents about how they envision their choice of strategies will address determinants to shed more light on the selection process [33]. We recommend that researchers explicitly specify hypothesized causal pathways through which implementation strategies exert their effects [35, 37] and specify implementation strategies using established reporting guidelines [3, 38]. Both of these steps will move researchers toward a greater understanding of when, where, how, and why implementation strategies are effective in improving implementation outcomes.

Systematic approaches to identifying determinants and matching strategies to address them have been developed [9, 20, 35, 39]. One way to address differences in assumed mechanisms and implied pathways of change, as highlighted above, is to use a transparent process to systematically and consistently match implementation strategies to address barriers such as intervention mapping (IM) [20]. IM has been recognized as a useful method for planning multifaceted implementation strategies [9, 37, 40]. A key feature of IM since its inception [41] has been its utility for developing multifaceted implementation interventions to enhance the adoption, implementation, and maintenance of clinical guidelines [42] and EBIs [43–49].

IM guides the development or selection of strategies by (1) conducting or using results from a needs assessment to identify determinants (barriers and facilitators) to implementation and identifying program adopters and implementers; (2) describing adoption and implementation outcomes and performance objectives (specific tasks related to implementation), identifying determinants of these, and creating matrices of change objectives; (3) choosing theoretical methods (mechanisms of change [19]); and (4) selecting or designing implementation strategies (i.e., practical applications) and producing implementation strategy protocols and materials. The detailed steps in this process help explain how the range of potential assumptions made by respondents as they chose strategies to address CFIR determinants in the present study may have contributed to the general lack of consensus in choices.

Within IM, identifying a barrier is not sufficient to guide the choice of a strategy. To thoroughly understand a problem with enough specificity to guide the effective selection of discrete strategies, the causes of each barrier must be specified along with the desired outcome in very specific terms (i.e., performance objective for implementation and their determinants). It is then necessary to identify specific methods or techniques [19, 20, 50] that can influence these determinants and operationalize these methods into concrete strategies. Behavior change techniques and methods (which stem from the basic sciences of behavior, organizational, and policy change) then are transparently and clearly specified within each selected implementation strategy.

Most intervention developers who follow systematic processes to create complex interventions at multiple levels would not select strategies (even strategies that had been widely used and clearly defined) without careful planning to ensure that the strategies would effectively address determinants of the problem or desired outcome. Ratings of considerations that most influenced the selection of strategies are consistent with this. Relevance and improvement opportunity were the primary influences on the rankings, and these considerations are also integral to the first three steps of IM noted above. Feasibility was also influential, but it did not seem to overshadow the need for a careful evaluation of the needs of the initiative through a process like IM. The work and resource requirements of an implementation strategy (i.e., difficulty) were influential, but not as influential as the other considerations. This may reflect the assumption that if an implementation initiative is a priority, work and resource requirements will be allocated to address barriers to implementation.

In implementation science and practice, a process akin to IM (systematically identifying barriers, determinants of these, change methods for addressing them, and development or selection for specific strategies) is not often followed or clearly documented, leading to gaps in understanding which strategies work and why they produce their effects. Implementation science is a relatively young field and frameworks to help identify implementation determinants such as CFIR have only recently been developed and operationalized. Nonetheless, exemplary studies can be found [49]. For example, Garbutt and colleagues utilized CFIR to characterize contextual determinants of EBI use, analyzed those determinants systematically via a theoretical framework, identified specific behavior change targets, and then selected relevant implementation strategies [51].

### Limitations

This study has several limitations that may impact the validity of the inferences that can be made from this data set. In the absence of consensus in the field regarding how to identify “implementation experts,” this project asked individuals to self-identify whether or not they are implementation experts based upon a practical definition (“Implementation experts have knowledge and experience related to changing care practices, procedures, and/or systems of care. Based on the above definition, could someone accuse you of being an implementation expert?”). The breadth of this definition aimed to be inclusive of both researchers and practitioners who primarily work in the field. However, 81% of respondents indicated that research consisted of more than half of their job duties. Thus, different results may have been elicited if more non-research practitioners had been recruited. It is also possible that relying upon more objective indicators of expertise (e.g., individuals’ implementation science and practice-focused funding, publications, or relevant service on editorial boards or expert advisory committees) may have yielded a different pool of participants and different results.

As noted previously, the diversity of endorsements linking specific implementation strategies to specific barriers may reflect the diverse assumptions respondents brought to the task regarding how barriers interact with the program-specific needs given respondents’ unique histories of implementing different innovations. Given the abstract characterizations of both the barriers and the strategies, gathering data on these assumptions may have produced further insights into the endorsements provided. Future projects focusing on the implementation of specific innovations would benefit from adopting two practices. First, when a specific innovation is being investigated, it is possible to more clearly operationalize implementation strategies as they are applied to that innovation, using established reporting guidelines to specify the details of each strategy and articulate how they address contextual barriers [3, 38]. Second, within the specific initiative, respondents can specify the causal pathways through which included implementation strategies are hypothesized to exert their effects [35, 37].

## Conclusions

Self-identified implementation experts engaged in a process to select up to seven ERIC implementation strategies that would best address specific CFIR-based barriers. Respondents’ endorsements formed the basis for the CFIR-ERIC Implementation Strategy Matching Tool. This tool can serve as a preliminary aid to implementers and researchers by supporting consideration of a broad array of candidate implementation strategies that may best address CFIR-based barriers. Due to the heterogeneity of endorsements obtained in this study, the ranked considerations provided by this tool should be coupled with a systemic process, such as intervention mapping, to further develop or identify and tailor strategies to address local contextual determinants for successful implementation.

## Additional file

Additional file 1: The level of endorsement for each of the 2847 possible individual barrier-strategy combinations (XLSX 50 kb)

## Abbreviations

CFIR: Consolidated Framework for Implementation Research
EBI: Evidence-based innovation
ERIC: Expert Recommendations for Implementing Change
HCV: Hepatitis C virus
IM: Intervention mapping
NIRN: National Implementation Research Network
SIRC: Society for Implementation Research Collaboration
TLC: Telephone lifestyle coaching
VHA: US Veterans Health Administration
